# Structural Design and Crawling Pattern Generator of a Planar Quadruped Robot for High-Payload Locomotion

**DOI:** 10.3390/s20226543

**Published:** 2020-11-16

**Authors:** Ru Kang, Fei Meng, Xuechao Chen, Zhangguo Yu, Xuxiao Fan, Aiguo Ming, Qiang Huang

**Affiliations:** 1 Intelligent Robotics Institute, School of Mechatronical Engineering, Beijing Institute of Technology, Beijing 100081, China; kangru@bit.edu.cn (R.K.); chenxuechao@bit.edu.cn (X.C.); yuzg@bit.edu.cn (Z.Y.); yuyan0672@163.com (X.F.); ming@mce.uec.ac.jp (A.M.); qhuang@bit.edu.cn (Q.H.); 2 The Beijing Advanced Innovation Center for Intelligent Robots and Systems, Beijing Institute of Technology, Beijing 100081, China; 3 Department of Mechanical Engineering and Intelligent Systems, The University of Electro Communications, Tokyo 182-8585, Japan

**Keywords:** quadruped robot, load capacity, crawling, trunk swaying

## Abstract

Load capacity is an important index to reflect the practicability of legged robots. Existing research into quadruped robots has not analyzed their load performance in terms of their structural design and control method from a systematic point of view. This paper proposes a structural design method and crawling pattern generator for a planar quadruped robot that can realize high-payload locomotion. First, the functions required to evaluate the leg’s load capacity are established, and quantitative comparative analyses of the candidates are performed to select the leg structure with the best load capacity. We also propose a highly integrated design method for a driver module to improve the robot’s load capacity. Second, in order to realize stable load locomotion, a novel crawling pattern generator based on trunk swaying is proposed which can realize lateral center of mass (CoM) movement by adjusting the leg lengths on both sides to change the CoM projection in the trunk width direction. Finally, loaded crawling simulations and experiments performed with our self-developed quadruped robot show that stable crawling with load ratios exceeding 66% can be realized, thus verifying the effectiveness and superiority of the proposed method.

## 1. Introduction

The design of bionic robots has benefited from the introduction of robust and energy-saving movements based on those of animals. When compared with wheeled robots and other types of legged robots, quadruped robots can perform in response to external stimulation in an accurate and ideal manner of conditioned reflection and have the natural advantages of trafficability and agility on complex outdoor terrain [1,2]. At the same time, these robots offer further advantages in terms of dynamic locomotion, stability and manufacturing cost. Many advanced quadrupeds with superior motion performance are available, and stability and dynamic motion ability of these robots have developed rapidly [3,4,5]. As the key performance index of legged robots, the load capacity has important research significance to improve robots’ practicability, such as in material transportation and in rescue efforts in dangerous environments. When the driving mode is kept the same, the main factors affecting the payloads of legged robots are the load capacity of the structure and the stability of movement.

The leg mechanism plays an extremely important role in both the kinematic and dynamic characteristics and has always been one of the most important issues in quadruped robot performance research, along with high speed performance, mobility and payload capacity [6,7]. Most of the representative robots have used a series leg structure, which is characterized by the combination of a simple structure and large workspace; robots to use this structure include BigDog [8], HyQ [9], SCalf [10], ANYmal [11] and MIT Cheetah 3 [12]. As an alternative, some planar quadruped robots can realize pronking and jumping using parallel legs with two degrees of freedom (DoF), which combines the advantages of a large load capacity and good structural stability; robots of this type include Minitaur [13], Staford Doggo [14] and SpaceBok [15]. A hydraulic four-legged walking machine was proposed characterized by a high payload capacity, with its legs comprising a new family of parallel mechanisms [16,17], but this robot structure was complex and its weight was large (130 kg). Although different leg types have been widely developed and used in quadruped robots, studies have mainly focused on dynamic and agile movement; there has been little research into the design method of leg structures with a high payload.

In addition, to improve the stability of load locomotion, a force distribution approach of joint space distribution was proposed to promote the payload capability of hexapod robots with parallel leg mechanisms [18]. A hexapod robot with a high payload–weight ratio was proposed by deriving the optimized objective function from the perspective of dynamic behaviors [19]. However, most research works have been aimed at hexapod robots, and there is little research on quadruped robots. One type of stable gait, called crawling, which was introduced by McGhee, is frequently used by mammals during loaded locomotion [20]. The most stable and widely used walking standard is based on the zero moment point (ZMP) theory [21], which can ensure that the planned CoM position always lies within the ZMP stable area. This method is mainly used for biped robots, and several walking pattern generators have been developed [22,23]; some studies have also applied this method to quadruped robots [24,25,26]. According to the stability margin and related ZMP theory, the center of the supporting polygon is the most stable point, and the support triangle switching of a quadruped robot’s crawling process will produce a displacement in the trunk width direction. However, it is difficult to realize the lateral motion of the CoM of planar quadruped robots which use most of the existing planning methods, because they lack a lateral swaying DoF. There has also been little research into the planning method of crawling locomotion for planar robots (the gait with the best load capacity).

In summary, few studies have aimed to improve the load performance of robots in terms of their structural design and control method from a systematic point of view. To overcome the deficiencies described above, a structural design method and crawling pattern generator for a planar quadruped robot with a high payload is proposed in this paper. This robot is composed of four parallel legs, which have the advantages of a superior load capacity and good structural stability. Each parallel leg only needs two DoFs, as fewer DoFs reduce the number of driving modules and the weight, as well as making the structural design simpler. The main contributions of this paper are as follows:

(1) Based on the accurate definition of leg structure type, evaluation functions to measure the leg-load capacity and the joint motion performance of the robots are established; furthermore, quantitative comparative analyses are carried out to select the leg structure with the best payload. A design method for a highly integrated driver module is also proposed which reduces the module size and the leg mass; the spatial layout of the symmetrical outer span of the U-frame improves its load capacity and impact resistance.
(2) To realize stable high-payload locomotion for a quadruped robot with the fewest DoFs, a novel crawling pattern generation method based on trunk swaying is proposed. Using this method, lateral CoM movement is realized by adjusting the leg lengths on both sides, which can change the CoM projection to track the ZMP in the trunk width direction; a feedforward and feedback control method based on trunk position compliance (TPC) is also proposed to provide further stability improvements.

This paper is organized as follows. The structural design is introduced in Section 2. In Section 3, the crawling pattern generator for a planar quadruped robot based on trunk swaying is proposed. Section 4 presents the numerical simulations and physical experiments performed to verify the proposed method. Section 5 concludes this paper and proposes future work.

## 2. Structural Design

The leg mechanism plays an important role in the kinematic and dynamic characteristics of robots [6,11], and its mechanism forms the performance research basis of quadruped robots, including their payload capacity and movement stability. Therefore, to improve the payload capacity, it is essential to design a high-payload leg structure and suitable spatial layout for the driver modules.

### 2.1. Leg Structure Selection via Quantitative Analysis

In this section, an accurate definition of leg structure types is given according to leg rods’ driving relationship; furthermore, the functions required to evaluate the leg load capacity are established and quantitative comparative analyses of the candidates are performed to select the leg structure with the best load capacity. The workspace and joint motion performance of different legs are also analyzed.

#### 2.1.1. Definition of Leg Structure Type

**Figure 1:**
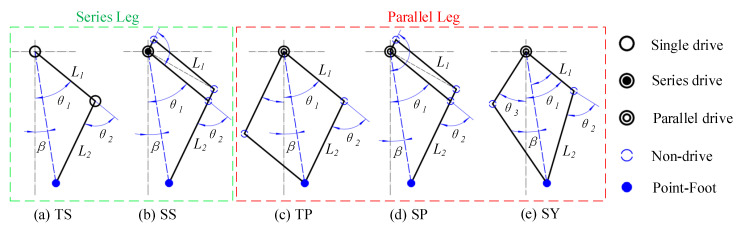
Different leg structures. (**a**) Traditional series (TS), (**b**) special series (SS), (**c**) traditional parallel (TP), (**d**) special parallel (SP) and (**e**) symmetrical (SY). The black hollow circle represents a single drive, the black circle containing a solid point represents two series drives oriented along the same axis, the black circle containing a hollow circle represents two parallel drives along the same axis, the blue hollow circle represents no drive and the blue solid point represents the point-foot.

Leg structure is defined based on a process of summarizing multiple driving distributions in this work. Using the example of a single leg with two DoFs, if a knee motor stator is assembled on the hip motor rotor or on any components driven by the hip rotor, the position of the knee joint motor will be changed when the hip joint motor rotates; this configuration relationship is called the series leg. In contrast, if the motor stators for the knee and hip joints are assembled on the same fixed component, the position of the next motor will not be affected when the previous motor operates; this configuration relationship is called the parallel leg. The classifications are shown in detail in Figure 1. The series leg is shown in the green dotted box, including both the traditional series (TS) and special series (SS) structures, and the parallel leg is shown in the red dotted box, including the traditional parallel (TP), special parallel (SP) and symmetrical (SY) structures.

#### 2.1.2. Workspace

The workspace is an important motion constraint for legged robots and us mainly determined by two factors: the leg length *l* (i.e., the distance from the hip joint to the end of the foot) and the swaying range of tge leg, which are both dependent on the installation forms and the spatial distributions of actuators. ${ℜ}^{2}$ was used to represent the complete set of plane points (*l*, β(l)) that can be reached by the two-dimensional leg structure, as shown in Equation (1):

(1) $${ℜ}^{2}\;=\;\left\{\left(l,\beta (l)\right)|l_{\min}⩽l⩽l_{\max}\right\},$$

where β(l) represents the angle of the whole leg relative to the reference coordinate system and $l_{\min}$ and $l_{\max}$ represent the minimum and maximum leg length, respectively.

To compare the workspaces of different legs, the area equivalent function is proposed to evaluate the workspace size, as shown in Equation (2):

(2) $$\begin{array}{cc} S_{\max}\left({ℜ}^{2}\right)=S\left(l,\beta_{\max}\right) & =\pi \left(l_{\max}^{2}-l_{\min}^{2}\right)\times \frac{\beta_{\max}}{2\pi }\\ \; & =\frac{\beta_{\max}}{2}\left(l_{\max}^{2}-l_{\min}^{2}\right), \end{array}$$

where $\beta_{\max}$ is the corresponding maximum swaying angle under the constraint of the different leg lengths.

As shown in Figure 1, the theoretical values of $l_{\max}$ and $l_{\min}$ of the different legs are all $L_{1}$ + $L_{2}$ and $|L_{1}-L_{2}|$, respectively. If the space of mechanical installation is unlimited, $l_{\min}$ is 0 when $L_{1}$ and $L_{2}$ are equal. When $L_{1}$ and $L_{2}$ are not equal, it is necessary to rotate the two driving rods over the horizontal plane that the hip joint axis passed to decrease the leg length, which will limit the workspace for the actual robot. The theoretical value of $\beta_{\max}$ in all cases is ($\theta_{1}$ + $\theta_{2}$). However, in actual mechanical structures, the swaying angle range of the parallel legs is smaller than that of the series structure because its foot end position is the result of the coupled motion of two driving rods that have specific constraints within the spatial structure, so the workspaces of the series legs (i.e., TS, SS) are maximal.

The workspace determines the spatial range that can be reached by the foot. A larger workspace provides greater flexibility and can meet the requirements of the robot’s special motion forms, so a larger workspace is preferable. However, the parallel leg structures have a sufficient amount of workspace to realize complete movement with a high load.

#### 2.1.3. Comparative Analysis of Load Capacity

Another important factor affecting the leg structure selection is load capacity. In the process of robot loading locomotion, the legs in the supporting phase need to provide enough supporting force in any preset swaying angle to realize continuous and stable walking. In combination with the quadruped robot’s loaded crawling requirements, the output torques of the hip joint (Joint 1) and the knee joint (Joint 2) under specific external loads are analyzed, allowing the candidate leg structures to be compared more intuitively. By deriving the forward kinematics of different leg structures, their foot positions can be expressed uniformly using the joint angle and length of leg rods, as shown in Equation (3):

(3) $$\left[\begin{array}{c} P_{X}\\ P_{Y} \end{array}\right]=\left[\begin{array}{cc} \sin\theta_{1} & \sin(\theta_{1}+\theta_{2})\\ -\cos\theta_{1} & -cos(\theta_{1}+\theta_{2}) \end{array}\right]•\left[\begin{array}{c} L_{1}\\ L_{2} \end{array}\right],$$

where $P_{X}$ and $P_{Y}$ are the foot positions in the X and Y directions, $L_{1}$ and $L_{2}$ are the lengths of the thigh and shank and $\theta_{1}$ and $\theta_{2}$ are the joint angles of the hip and knee, respectively, as shown in Figure 1.

Then, the relationship between the joint torque **τ** and the foot-end force ***f*** can be expressed uniformly as shown in Equation (4):

(4) $$\dot{P}=J\left(\Theta \right)\dot{\Theta },\;\tau \;=\;J{\left(\Theta \right)}^{T}f,$$

where J(Θ) is the Jacobian matrix.

In the TS and SS structures shown in Figure 1a,b, the hip joint angle and knee joint angle are both controlled directly by their respective motors, and the joint angles used in the Jacobian matrix are equal to the motor driving angle, so they can be used to calculate the joint output moment directly, as shown in Equation (5), where $J_{SS}=J_{TS}$, and the angular variables are $\theta_{1}$ and $\theta_{2}$.

(5) $$J_{SS}=J_{TS}=\left[\begin{array}{cc} \frac{\partial P}{\partial \theta_{1}} & \frac{\partial P}{\partial \theta_{2}} \end{array}\right]=\left[\begin{array}{cc} L_{1}\cos\theta_{1}+L_{2}\cos\left(\theta_{1}+\theta_{2}\right) & L_{2}\cos\left(\theta_{1}+\theta_{2}\right)\\ L_{1}\sin\theta_{1}+L_{2}\sin\left(\theta_{1}+\theta_{2}\right) & L_{2}\sin\left(\theta_{1}+\theta_{2}\right) \end{array}\right].$$

The knee joints of the TP and SP structures are not driven directly by the knee motor, as shown in Figure 1c–e, but are coupling-driven by the hip and knee joint motor. It is necessary to establish a fixed mapping relationship between the actual knee joint angle and the motor angle before using the latter to determine the output torque required. The Jacobian matrix for the TP and SP structures can be described as shown in Equation (6), where $J_{SP}=J_{TP}$, and the angular variables are $\theta_{1}$ and ($\theta_{1}$ + $\theta_{2}$).

(6) $$J_{SP}=J_{TP}=\left[\begin{array}{cc} \frac{\partial P}{\partial \theta_{1}} & \frac{\partial P}{\partial (\theta_{1}+\theta_{2})} \end{array}\right]=\left[\begin{array}{cc} L_{1}\cos\theta_{1} & L_{2}\cos\left(\theta_{1}+\theta_{2}\right)\\ L_{1}\sin\theta_{1} & L_{2}\sin\left(\theta_{1}+\theta_{2}\right) \end{array}\right].$$

Because of the structural characteristics of the SY leg, the relationship between the joint angle and the driving motor angle is more complex. The Jacobian matrix of the SY structure can then be described as shown in Equation (7), where the angular variables are $\theta_{1}$ and $\theta_{3}$.

(7) $$J_{SY}=\left[\begin{array}{cc} \frac{\partial P}{\partial \theta_{1}} & \frac{\partial P}{\partial \theta_{3}} \end{array}\right].$$

The relationship between the foot position and the leg length *l* and leg angle β was derived first; then, the relationship between *l*, β and the joint angles was established. The deduced process is shown in Equations (8)–(14):

(8) $$L_{s}=\sqrt{{L_{1}}^{2}+{L_{2}}^{2}+2L_{1}L_{2}\cos\theta_{2}},$$

(9) $$\beta =\theta_{1}-\arccos\frac{{L_{1}}^{2}+{L_{s}}^{2}-{L_{2}}^{2}}{2L_{1}L_{s}},$$

(10) $$\theta_{3}=2\beta -\theta_{1},$$

(11) $$J_{\mathrm{SY}}=\left[\begin{array}{cc} \sin\beta & L_{s}\cos\beta \\ -\cos\beta & L_{s}\sin\beta \end{array}\right]\left[\begin{array}{cc} {J_{\mathrm{SY}2}}^{11} & {J_{\mathrm{SY}2}}^{12}\\ {J_{\mathrm{SY}2}}^{21} & {J_{\mathrm{SY}2}}^{22} \end{array}\right],$$

(12) $${J_{\mathrm{SY}2}}^{11}=-\frac{L_{1}}{2}\sin\left(\frac{\theta_{1}-\theta_{3}}{2}\right)-\frac{{L_{1}}^{2}\sin\left(\theta_{1}-\theta_{3}\right)}{4\sqrt{\frac{{L_{1}}^{2}}{2}\left(\cos\left(\theta_{1}-\theta_{3}\right)-1\right)+{L_{2}}^{2}}},$$

(13) $${J_{\mathrm{SY}2}}^{12}=\frac{L_{1}}{2}\sin\left(\frac{\theta_{1}-\theta_{3}}{2}\right)+\frac{{L_{1}}^{2}\sin\left(\theta_{1}-\theta_{3}\right)}{4\sqrt{\frac{{L_{1}}^{2}}{2}\left(\cos\left(\theta_{1}-\theta_{3}\right)-1\right)+{L_{2}}^{2}}},$$

(14) $${J_{\mathrm{SY}2}}^{21}={J_{\mathrm{SY}2}}^{22}=\frac{1}{2}.$$

#### 2.1.4. Comparative Analysis of Joint Motion Ability

To evaluate the robot’s manipulability and joint motion, standard manipulability measures were proposed [13,27]. In this paper, the evaluation function for the joint motion performance of the candidate leg structures was improved: $\sigma_{\min}\left(J\left(\Theta \right)\right)$, the minimum singular value of the Jacobian matrix, which reflects the joint sensitivity (the minimum foot-end velocity to a unit joint velocity) and can be obtained using Equation (15); thus, a higher value of $\sigma_{\min}\left(J\left(\Theta \right)\right)$ is more conducive to an enhanced joint drive capability.

(15) $$\begin{array}{cc} \sigma_{\min}\left(J(\Theta )\right) & =\min_{\left\|f=1\right\|}\sigma^{\frac{1}{2}}\left(f^{-T}\tau^{T}\tau f^{-1}\right)=\min_{\left\|f=1\right\|}\sigma^{\frac{1}{2}}\left(\tau^{T}\tau \right). \end{array}$$

$\sigma_{\max}\left(J\left(\Theta \right)\right)$, which is the maximum singular value of the Jacobian matrix, reveals the maximum impact force on the actuator caused by a unit motor torque and can be obtained using Equation (16); thus, a lower value of $\sigma_{\max}\left(J\left(\Theta \right)\right)$ is preferred.

(16) $$\begin{array}{cc} \max_{\left\|\tau =1\right\|}\|f^{T}f{\|}_{2} & \;=\;\max_{\left\|\tau =1\right\|}\|\tau^{T}J{\left(\Theta \right)}^{-1}J{\left(\Theta \right)}^{-T}\tau {\|}_{2}\;=\;\frac{1}{\sigma_{\max}^{2}\left(J(\Theta )\right)}. \end{array}$$

Based on the above analysis, the most suitable leg structure for high-payload locomotion was selected by considering the workspace, load capacity and joint motion performance comprehensively. Therefore, the simulation settings were as follows: 100 N loads (about a quarter of the robot’s weight) were fixed to one leg and the corresponding output torques of the motors in the same reachable workspace were calculated. Additionally, the joint performance was analyzed depending on the evaluation functions.

**Figure 2:**
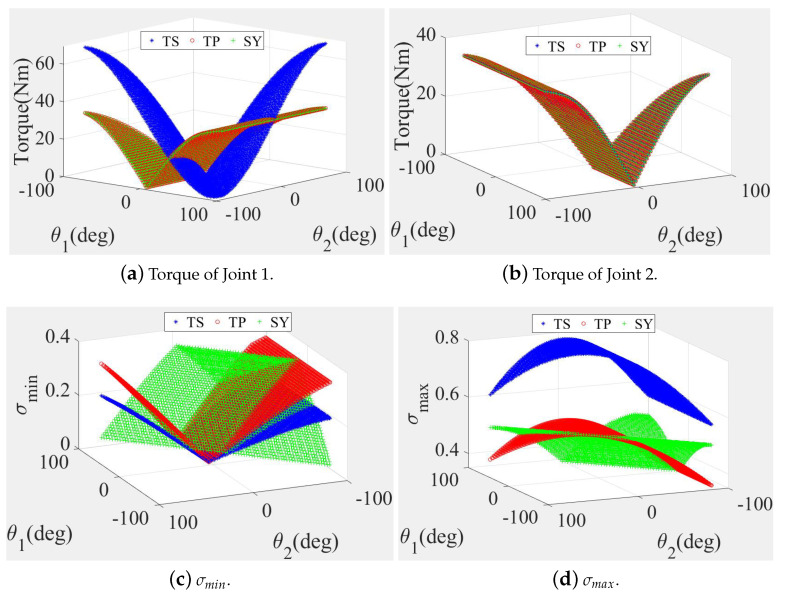
Joint torques and motion performance comparisons of three legs (TS, TP and SY) when $L_{1}$ = $L_{2}$.

The calculated results show that when $L_{1}$ = $L_{2}$, the output torques of Joint 2 for the three candidate leg structures were exactly equal, as shown in Figure 2b. However, the output torque of Joint 1 of the TS structure was much higher than that of the other structures in the larger workspace, as shown in Figure 2a. The $\sigma_{\min}$ values of the TP and SY structures were high and the $\sigma_{\max}$ values of the TP and SY structures were low, as shown in Figure 2c,d.

**Figure 3:**
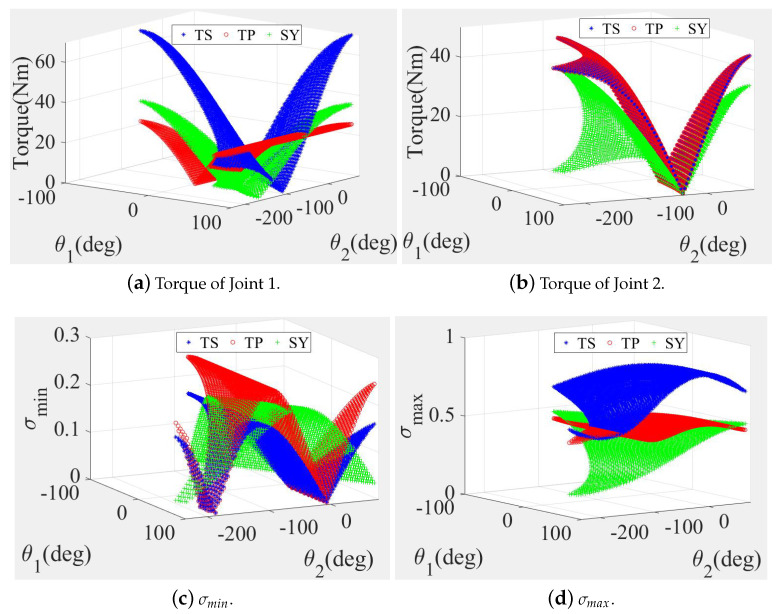
Joint torques and motion performance comparisons of three legs (TS, TP and SY) when $L_{1}$<$L_{2}$.

In addition, when $L_{1}$<$L_{2}$, the output torques of Joint 1 of the TP and SY structures were much lower than that of the TS structure in most of the workspaces, as shown in Figure 3a. The change trends in the Joint 2 output torques of these two structure types were similar, as shown in Figure 3b. Furthermore, the torques of both Joint 1 and Joint 2 of the SY structure were lower than that of the TP structure, which could provide energy savings and a higher theoretical working efficiency. However, it is difficult to establish an accurate model of the transmission from the motor output shaft to the actual mechanical actuator. If the theoretical output torque were to be smaller than the system damping ratio of the transmission structure, it would then be difficult to control the joint motion accurately. Besides, both the $\sigma_{\min}$ and $\sigma_{\max}$ values of the TP and SY structures were also favorable, as illustrated in Figure 3c,d, respectively.

The calculations and discussions above indicate that the TP structure has a greater load capacity and offers superior joint motion performance in a larger proportion of the workspace, which makes it more suitable for a loaded crawling robot. Therefore, TP legs were selected to configure a quadruped robot with a high payload.

### 2.2. Integrated Design of Driver Module

The assembly relationship between the driving module, main support pedestal, leg rods and driving flanges affects the workspace and load capacity directly. To maximize the workspace, the Stanford Doggo [14] had two actuator modules installed on its torso in the form of a suspension to allow its entire leg to rotate within the full angular range, but this structure places high demand on the strength of the suspension shaft, which cannot withstand high-strength impacts. The other leg structures have shown similar characteristics; the pursuit of a larger workspace requires a compromise in terms of the load capacity of the leg structure.

**Figure 4:**
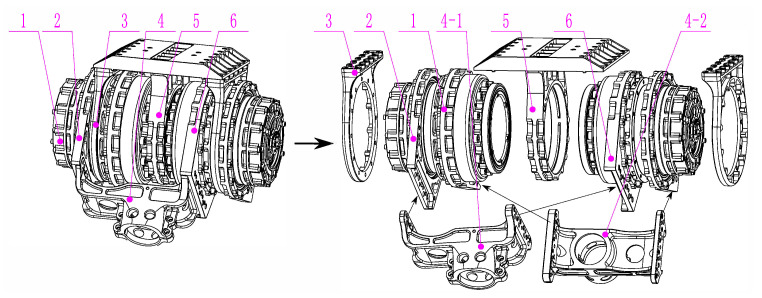
Highly integrated design of driver module. 1. Driving module, 2. driving flange, 3. side support, 4. output U-frame, 5. main support pedestal, 6. ancillary shoring flange.

To improve the load capacity of the structure under the premise of a sufficient range of motion, a highly integrated design method for a driver module is proposed in this paper. The left image in Figure 4 shows the complete assembly structure of the integrated driver module, while the right image shows the exploded view. Two driving modules that integrate a motor and a reducer were installed on the two sides of the main support pedestal, and they were both coaxial and symmetrical. The actuators are required to generate enough torque and speed to satisfy the loading demand. Taking these factors into account, a self-developed motor was selected to drive the joints. Its power density was 808.82 W/kg, which corresponded to the top level in the same specification [28]. In addition, when the reduction ratio was increased, the output torque became larger and the speed was reduced. However, a high-ratio reducer may cause a great deal of rotational inertia and decrease the transparency of the actuator. Thus, a harmonic reducer with a reduction ratio of 31:1 was selected by considering both the output torque and transparency during load movement.

The two driver modules were fully fixed using two side supports and the main support pedestal, with one leg rod being driven by each driver module. Another design was proposed to avoid the asymmetrical force caused by the cantilever driving mode, in which the output U-frame was designed as shown in Figure 4; one side of the U-frame was connected to the driving flange of the driver module, while the other side of the frame was connected to the ancillary shoring flange installed at the outer edge of another driver module, and another U-frame had the same connection. The two driving leg bars were connected to the circular hole of two U-frames, respectively. Because the driver modules, U-frame mechanism and the connecting parts were all distributed symmetrically in space, the U-frame mechanism was able to distribute the driving force symmetrically to the actuators.

This layout can divert the external impact to the shells of driver modules with a much larger diameter rather than being borne directly by the drive output module. The impact resistance is greatly improved and the entire module size and weight are greatly reduced while allowing both uniform transmission and force diffusion to be realized. Therefore, this driver module improves the load capacity while reducing the self-weight of the robot, which can increase the robot’s payload greatly.

### 2.3. High-Payload Quadruped Robot

**Figure 5:**
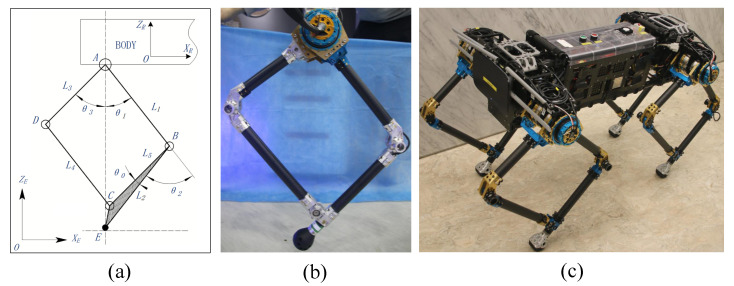
Single leg structure and overview of quadruped robot. (**a**) Accurate modeling of TP single leg, (**b**) TP single leg, (**c**) planar quadruped robot.

In order to realize a higher payload, this paper proposes a quadruped robot based on the leg structure selection and the spatial layout design of driver modules shown in Figure 5c.

For our quadruped robot, we selected TP legs with two DoFs, which reduced the number of driving modules and the system weight and also made the structural design simpler. The passive rotation joint was set outside the leg bar to improve the latter’s strength and reliability, and the ABCD structure formed a parallelogram, as shown in Figure 5a. Complete motion of a single leg was realized by the coupled rotation of rods AB and AD around point A, while rods BC and CD were driven passively, and the triangle BCE moved along rod BC as a fixed structure. The actual parallel leg was made from a light carbon fiber and a high-strength aluminum alloy and is shown in Figure 5b. Elastic spherical polyurethane was used for the robot’s foot to improve its buffering performance.

The control system of our robot consists of an Industrial Personal Computer (IPC) as an upper computer to execute real-time planning and information processing and an Elmo Motor Driver as a lower controller to support the underlying position control of motors. Between the IPC and drivers, a CAN bus was adopted as a transmission mode [28]. The robot has eight active joints, and each of these joints is equipped with an incremental encoder (RMB20IC). A six-axis inertial measurement unit (IMU-MTi100) was mounted at the center of the trunk to measure the posture and acceleration of the robot. One three-dimensional force sensor was located under each foot to measure the reaction force from the ground.

The trunk height (leg unfolded) and length of the robot are 75 cm and 80 cm, respectively. The total robot mass (including all sensors and controllers) is approximately 30 kg, the weight of each single leg is mainly concentrated in the upper part and the mass ratio of the leg bar to the whole robot is less than 0.18; this reduces the leg’s moment of inertia during motion, which causes the motion model to be closer to the linear inverted pendulum model (LIPM). Therefore, the accuracy of the motion model is initially improved compared to that of the hardware. Our self-developed planar quadruped robot has achieved trotting, bounding and pronking gaits and has strong dynamic movement abilities.

## 3. Crawling Pattern Generator Based on Trunk Swaying

**Figure 6:**
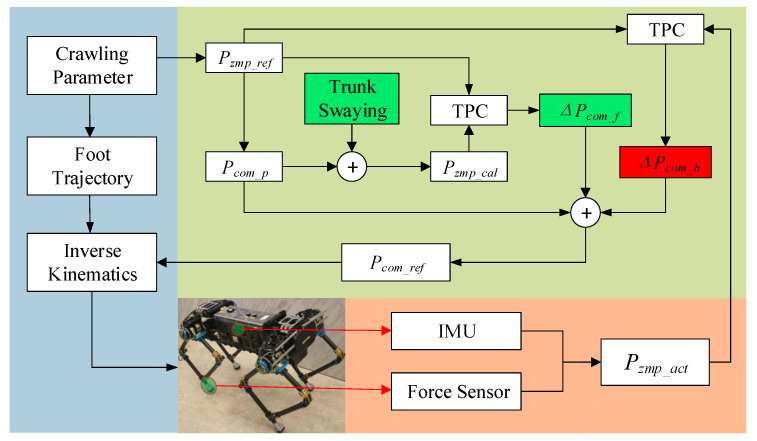
Crawling pattern generator based on trunk swaying. $P_{zmp\_ref}$ is the planned zero moment point (ZMP), which acts as the reference; $P_{com\_p}$ is the pre-theoretical center of mass (CoM) calculated based on $P_{zmp\_ref}$; $P_{zmp\_cal}$ is the calculated ZMP based on $P_{com\_p}$ and trunk swaying; $\Delta P_{com\_f}$ is the feedforward term of the CoM position based on trunk position compliance (TPC); $P_{zmp\_act}$ is the actual ZMP during real-time crawling according to the inertial measurement unit (IMU) and force sensors; $\Delta P_{com\_b}$ is the feedback term of the CoM position based on $P_{zmp\_act}$; and $P_{com\_ref}$ is the CoM reference trajectory.

It was necessary to ensure that the robot system operates within its set stability margin in each walking cycle. To realize stable loaded crawling for the planar quadruped robot, a novel crawling pattern generation method based on trunk swaying is proposed in this section. The overall framework of the crawling pattern generator is shown in Figure 6.

The CoM trajectory is calculated using an analytical method that is accurately based on the LIPM; then, the CoM lateral movement is generated via trunk swaying by adjusting the leg lengths on both sides. In addition, the theoretical ZMP deviation caused by the trunk swaying and the actual ZMP deviation were compensated with reference to the CoM trajectory as feedforward and feedback terms, respectively.

### 3.1. CoM Trajectory Generation Based on LIPM

When quadruped robots are faced with larger loads, they usually adopt a crawling gait, in which one leg at most is swaying at any time and at least three of its legs are supporting. There is always a support area, which allows quadruped robots to maintain their static stability. The stability margin and the related ZMP theory indicate that the center of the supporting triangle is the most stable point. The swaying order of the four legs in the crawling gait is as follows: RF (right front)–LH (left hind)–LF (left front)–RH (right hind). This order can be summarized as follows: after the front leg sways, the diagonal hind leg then sways, and the front leg on the same side sways after the hind leg.

**Figure 7:**
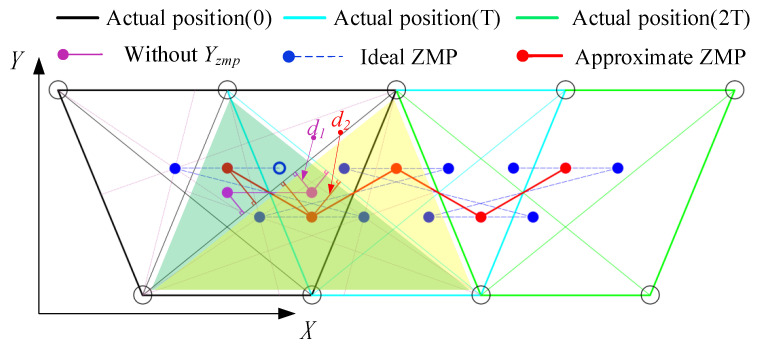
Evolution process of the ZMP trajectory. Black, cyan and green lines represent the support polygons of the quadruped robots at 0, T and 2T, respectively.

Based on this swaying law, the ZMP changes that occurred at each step for two cycles were analyzed as shown in Figure 7. The magenta solid point represents the planning ZMP without $Y_{zmp}$, and $d_{1}$ represents the minimum distance between the ZMP and the support edge. The blue line that connects the solid blue circles represents the ideal ZMP curve based on the most stable ZMP region. It is easy to determine that the ZMP will bounce back and forth during forward motion. To realize stable locomotion, the ZMP trajectory was approximated by replacing the blue line with the red line, ensuring that the ZMP remained unchanged during the swaying of the ipsilateral leg, and the corresponding minimum distance between the ZMP and the support edge was found to be $d_{2}$. It is obvious that $d_{2}$>$d_{1}$, and so the latter is more stable.

In this paper, our robot used the TP legs, which were able to integrate more weight, such as the driver module, into the hip joint near the CoM. The entire body could thus be virtually equivalent to a single-mass LIPM, with the center of each triangle formed by the three supporting legs acting as the origin; the two-dimensional motion within the XOZ plane was mainly analyzed. To simplify the required calculation process, the effects of the robot’s low angular speed were ignored when crawling initially; thus, the simplified dynamic model was as shown in Equation (17):

(17) $$m_{c}{\ddot{x}}_{c}z_{c}-m_{c}\left(g-{\ddot{z}}_{c}\right)\left(x_{c}-x_{zmp}\right)=0,$$

where $m_{c}$ is the robot mass, ${\ddot{x}}_{c}$ and ${\ddot{z}}_{c}$ are the accelerations and $x_{c}$ and $z_{c}$ are the positions in the *X* and *Z* directions, respectively, and $x_{zmp}$ is the ZMP position in the *X* direction. The dynamic model in the *Y* direction is similar to that in the *X* direction.

**Figure 8:**
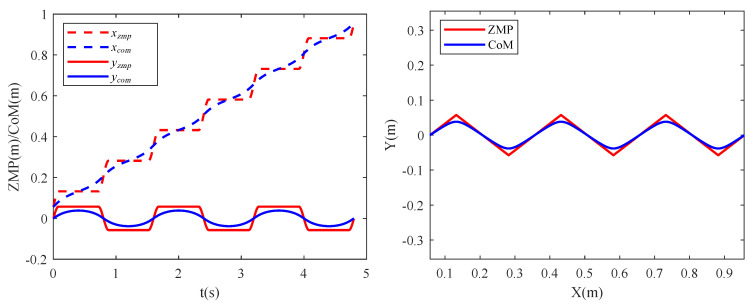
ZMP and CoM trajectory in three cycle. The (**left**) image shows the ZMP and CoM trajectories in *X* and *Y* directions, respectively. The (**right**) image shows the composite motion in the XOY plane.

As shown in Figure 8, the smooth ZMP trajectory was fitted using the piecewise functions of cubic polynomials, and an analytical solution for the CoM trajectory was then obtained by solving Equation (8). To achieve a better ZMP transition, a quadruped support phase was added after the swaying of the RF and LF legs.

### 3.2. Realization of Lateral Movement Based on Trunk Swaying

In general, omnidirectional motion generation for a legged robot mainly involves initially generating the motion trajectory in two directions and performing an orthogonal synthesis of the motion in two directions. This method is easy to implement for omnidirectional DoF robots. However, for planar robots, the lateral motion cannot be generated directly because of the lack of lateral swaying DoF.

**Figure 9:**
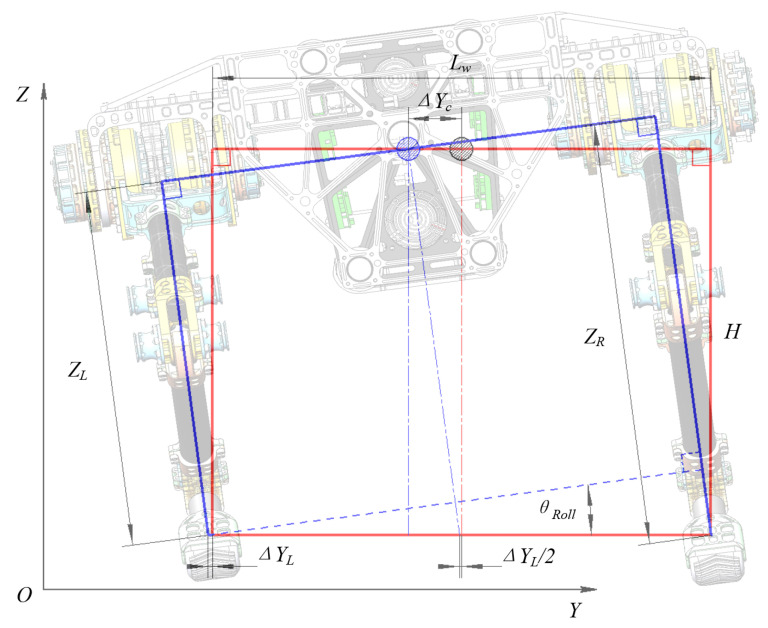
Rear view of a planar quadruped robot with trunk swaying. The red solid line represents the original posture, the blue solid line represents the posture after trunk swaying, and the circle with the section line represents the CoM position.

To solve this problem, an innovative lateral motion generation method based on trunk swaying is proposed. In this method, the CoM position moves along the *Y* direction, and its height must always remain constant. The original leg length is *H* and the trunk is horizontal, as indicated by the red line in Figure 9.

The hip joint is connected to the trunk in a fixed manner; therefore, to cause the CoM to produce a left $\Delta Y_{c}$ offset, the CoM projection in the *Y* direction was changed while the foot position remained unchanged. If we want to make the CoM move by $\Delta Y_{c}$ to the left, this can be achieved by adjusting the lengths of the left and right legs from *H* to $L_{L}$ and $L_{R}$, respectively. It should be noted here that all legs are always oriented perpendicular to the horizontal plane of the robot’s trunk. Therefore, the trunk roll angle will change from 0 to $\theta_{Roll}$, which causes the CoM projection in the *Y* direction to change. The joint angle sequence can be determined by solving the inverse kinematics of the CoM trajectory and the foot trajectory. The required movements in two directions can be realized by changing the leg lengths at the same time using this method. Based on the planned value of $Y_{zmp}$, the lengths of the left and right legs at each moment can be calculated using Equations (18) and (19), respectively:

(18) $$L_{L}=\sqrt{H^{2}+\Delta {Y_{c}}^{2}}-\frac{L_{w}\times \Delta Y_{c}}{2H},$$

(19) $$L_{R}=\sqrt{H^{2}+\Delta {Y_{c}}^{2}}+\frac{L_{w}\times \Delta Y_{c}}{2H},$$

where $L_{L}$ and $L_{R}$ represent the lengths of the left and right legs, respectively, $L_{w}$ represents the trunk width (i.e., the distance between the left and right hip joints) and $\Delta Y_{c}$ represents the CoM offset in the *Y* direction ($\Delta Y_{c}$ = $Y_{zmp}$).

Then, $L_{L}$ and $L_{R}$ were inserted into the inverse kinematics formulas to plan the crawling motion. In this paper, it is stipulated that the foot relates to the global coordinate system; i.e., the hip joint height always remains unchanged (which is given by $Z_{H}$ = *H*) when the forward motion is generated. Therefore, the leg length must be changed when the lateral motion is generated, and the hip joint position in the *Z* direction must also be changed when the foot end position remains unchanged; i.e., $Z_{H}$ = $L_{L}$ or $L_{R}$.

### 3.3. Crawling Stability Optimization

**Figure 10:**
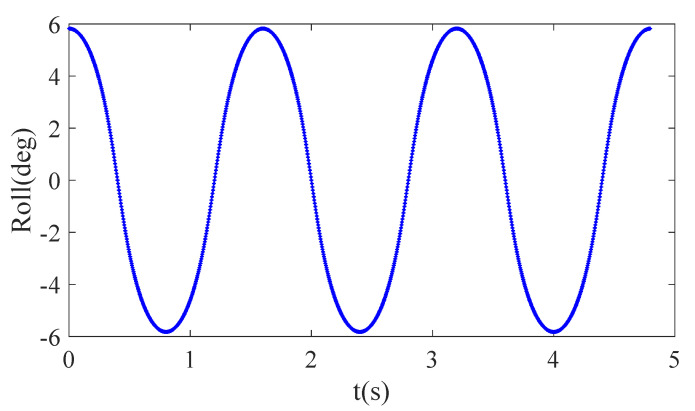
The roll angle curve in crawling motion.

The equations above show that the movement in the *Y* direction can be achieved by adjusting the leg length and that the distance to the foot end will increase; however, in the setting of the actual robot’s movement, it can be concluded that this distance, denoted by $\Delta Y_{L}$, is very small and can be ignored. Additionally, the roll angle of the robot’s trunk can be calculated during the process of tracking the CoM’s lateral trajectory using Equation (20), and the roll angle will change repeatedly during the crawling process, as shown in Figure 10.

(20) $$tan\theta_{Roll}=\frac{L_{R}-L_{L}}{L_{w}}.$$

where $\theta_{Roll}$ represents the rotation angle of the robot system around the *X* axis.

However, the actual ZMP change caused by the higher rotational angular velocity cannot be ignored when the crawling speed increases. To enable crawling stability control, Japanese researchers proposed the TPC control method, which has proved to be quite effective for robots with highly rigid feet; this approach has been used in the humanoid robot H6 [29]. To simplify the required calculation process, the component due to the body’s moment of inertia was ignored when using the analytical method to solve the CoM trajectory, and the ignored component was then compensated in the CoM reference trajectory as the feedforward term $\Delta P_{com\_f}$, as shown in Figure 6. The $\theta_{Roll}$ generated during trunk swaying can be considered when calculating the theoretical ZMP in motion using Equation (21):

(21) $$x_{zmp\_cal}=\frac{m_{c}\left({\ddot{z}}_{c}+g\right)x_{c}-m_{c}{\ddot{x}}_{c}z_{c}-I_{y}{\ddot{\Omega }}_{y}}{m_{c}\left({\ddot{z}}_{c}+g\right)},$$

where $x_{zmp\_cal}$ is the calculated ZMP based on the original CoM trajectory, and $I_{y}$ and ${\ddot{\Omega }}_{y}$ are the moment of inertia and the angular acceleration around the *Y* axis, respectively.

The ZMP error caused by trunk swaying can be eliminated with the feedforward compensation term, $\Delta x_{f}$, which can be obtained by integrating $\Delta {\ddot{x}}_{f}$, which can be solved using Equation (22).

(22) $$\Delta {\ddot{x}}_{f}=-k_{1}\left(x_{zmp\_cal}-x_{zmp\_ref}\right)-k_{2}\Delta x_{f}-k_{3}\Delta {\dot{x}}_{f},$$

where $\Delta x_{f}$, $\Delta {\dot{x}}_{f}$ and $\Delta {\ddot{x}}_{f}$ represent the feedforward compensated CoM position, and its velocity and acceleration in the *X* direction, respectively. $k_{1}$, $k_{2}$, and $k_{3}$ are the coefficients of the state feedback gain, which can be calculated using a linear quadratic regulator. $x_{zmp\_ref}$ is the reference ZMP in the *X* direction.

To improve the robot’s stability during actual crawling, the actual ZMP can be calculated based on the results obtained from foot-force sensors and the IMU. The force on the foot end (${}^{R}F$) must be converted into the three-dimensional force (${}^{W}F$) relative to the global coordinate system using the coordinate conversion matrix (${}^{W}T_{R}$), as shown in Equations (23) and (24).

(23) $${}^{W}F{=}^{W}T_{R}\times^{R}F,$$

(24) $${}^{W}T_{R}=\left[\begin{array}{ccc} 1 & 0 & 0\\ 0 & \cos\alpha & -\sin\alpha \\ 0 & \sin\alpha & \cos\alpha \end{array}\right]\left[\begin{array}{ccc} \cos\theta_{2} & 0 & \sin\theta_{2}\\ 0 & 1 & 0\\ -\sin\theta_{2} & 0 & \cos\theta_{2} \end{array}\right],$$

where α is the roll angle of the robot relative to the global coordinate system measured by the IMU and $\theta_{2}$ represents the deflection angle of the force sensor relative to the robot trunk in the z-axis direction.

The actual ZMP calculation is then carried out, and the feedback compensation $\Delta x_{b}$ can be solved using Equations (25) and (26).

(25) $$x_{zmp\_act}=\frac{\sum_{i=1}^{4}\left(-{z_{fi}}^{w}F_{xi}+{x_{fi}}^{w}F_{zi}\right)}{\sum_{i=1}^{4}{}^{w}F_{zi}},$$

(26) $$\Delta {\ddot{x}}_{b}=-k_{1}\left(x_{zmp\_act}-x_{zmp\_ref}\right)-k_{2}\Delta x_{b}-k_{3}\Delta {\dot{x}}_{b},$$

where $x_{fi}$ and $z_{fi}$ are the positions of the *i*th force sensor in the *X* and *Z* directions, respectively. $F_{xi}$ and $F_{zi}$ are the force values of the *i*th force sensor in the *X* and *Z* directions, respectively. $\Delta x_{b}$, $\Delta {\dot{x}}_{b}$ and $\Delta {\ddot{x}}_{b}$ represent the feedback-compensated CoM position and its velocity and acceleration in the *X* direction, respectively.

The updated stable position of CoM can be obtained by $x_{com\_ref}$ = $x_{i}$ from Equation (27). In addition, the control approach above can be simplified to feedforward–feedback control (FFC).

(27) $$x_{i}={x_{i}}_{-1}+\Delta x_{f}+\Delta x_{b}.$$

In the equations above, the corresponding calculation processes for the *Y* direction are the same as those used for the *X* direction.

## 4. Simulations and Experiments

This section presents the simulations and experiments performed for the crawling motion of the quadruped robot based on the proposed pattern generator. The gait parameters and simulation settings are shown in Table 1.

### 4.1. Simulations

The quadruped crawling motion was simulated using V–REP dynamic software in this work. To evaluate the superiority of the proposed planning method more intuitively, comparative analyses of the CoM position errors ($\Delta P_{x}$ and $\Delta P_{y}$) were performed at different speeds. The stability margin is the most important parameter for quadruped locomotion. If the ZMP is located closer to the center of the stability region, the distance between the ZMP and the boundary of the support region will be larger and the robot’s motion will be more stable. Figure 7 shows the evolution of the stable region during the crawling process: the tracking errors between the actual ZMP and the theoretical ZMP ($\Delta x_{zmp\_act}$ and $\Delta y_{zmp\_act}$) become smaller, and the motion becomes more stable. In combination with the actual crawling speeds of quadruped robots, we mainly analyzed the positional errors and the ZMP tracking errors at three crawling speeds of 0.34 km/h, 0.68 km/h and 1.35 km/h.

**Figure 11:**
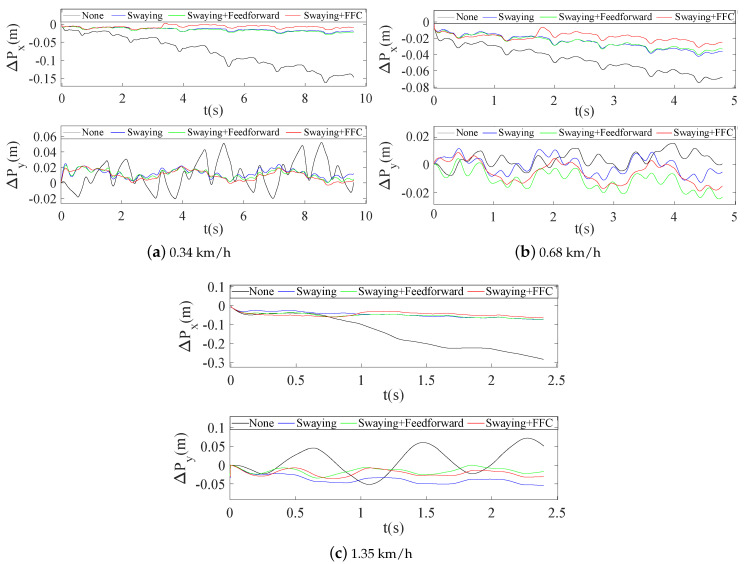
Trunk position errors at three different speeds.

As shown in Figure 11, $\Delta P_{x}$ without swaying showed major fluctuations and deviations (0.17 m, 0.07 m and 0.3 m, respectively) with increasing speed; however, these variations could be kept within a small fluctuation range when swaying and swaying + feedforward were used, and the tracking errors were further improved (0.02 m, 0.03 m and 0.06 m, respectively) when the proposed swaying + FFC approach was used. $\Delta P_{y}$ without swaying also showed major fluctuation ranges (−0.02 m to 0.05 m and −0.05 m to 0.08 m) at the speeds of 0.34 km/h and 1.35 km/h, respectively, but the fluctuations could again be kept within a small range (0.02 m, −0.02 m) when using the proposed method, and $\Delta P_{y}$ was maintained within a small range (−0.02 m to 0.015 m) at the speed of 0.68 km/h.

**Figure 12:**
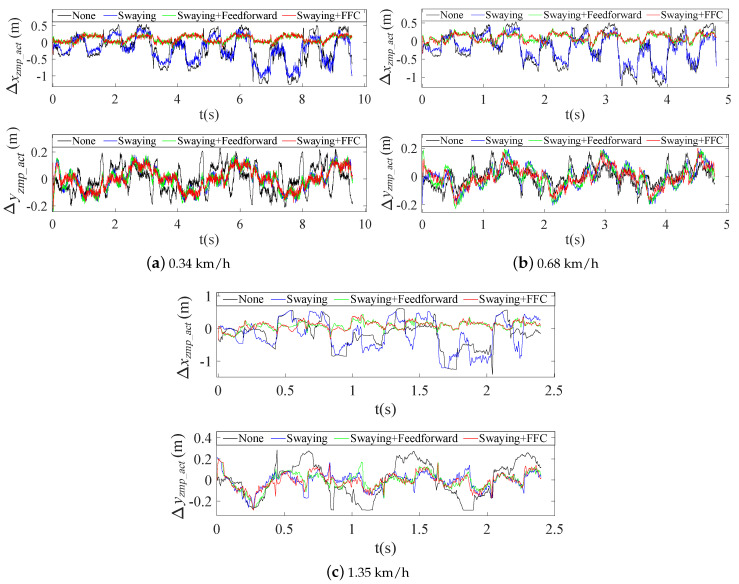
ZMP tracking errors at three different speeds.

As clearly shown in Figure 12, all the $\Delta x_{zmp\_act}$ values were within a large fluctuation range (−1.4 m to 0.6 m) when there was no swaying or swaying without compensation at the three speeds; however, the values were balanced within a small fluctuation range (−0.15 m to 0.3 m) when the FFC was added, as shown in Figure 12a–c. Additionally, the $\Delta y_{zmp\_act}$ values improved obviously when the swaying planning and FFC were added, and the errors changed their periodicity along with the crawling motion. The error was reduced to approximately half of the original value, especially at the higher speed of 1.35 km/h.

In addition, to verify the payload capacity of the robot, seven loads were set—i.e., no load, 5 kg, 10 kg, 15 kg, 20 kg, 25 kg and 30 kg—at the speed of 0.68 km/h. The ZMP errors were not obvious under the different loads according to the simulation results.

**Figure 13:**
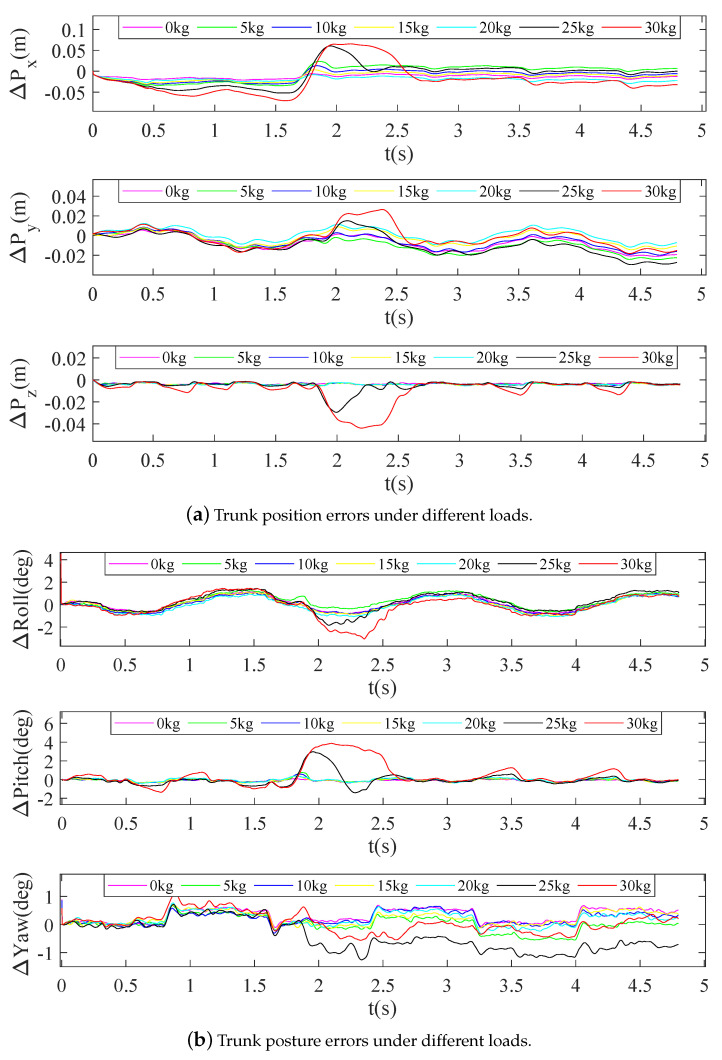
Trunk position errors and posture errors under different loads at the speed of 0.68 km/h.

Comparative analyses of the CoM position errors ($\Delta P_{x}$, $\Delta P_{y}$ and $\Delta P_{z}$) and the posture errors (ΔRoll, ΔPitch and ΔYaw) were used to evaluate the load crawling performance more intuitively, as shown in Figure 13. The $\Delta P_{y}$ values were all contained within a small region (−0.03 m to 0.03 m) under the different loads. The values of $\Delta P_{x}$ and the $\Delta P_{z}$ were also contained within small regions (−0.03 m to 0.02 m and −0.01 m to 0 m, respectively) when the load was less than 20 kg, but the values of these two parameters were at least double those of other values (−0.07 m to 0.06 m and 0 m to 0.04 m, respectively) when the load was more than 25 kg, as shown in Figure 13a. Additionally, ΔYaw showed different fluctuations (−1.2° to 1.2°) as load changed and followed no specific rule. The ΔRoll and the ΔPitch values were both within small fluctuation ranges (−1° to 1.2° and −1° to 1°, respectively) when the load was less than 20 kg, but varied within larger change ranges (−3° to 0° and 1.2° to 4°, respectively) when the load exceeded 25 kg, as shown in Figure 13b.

According to Figure 13, there was a fluctuation signature between about 2.0 s and 2.5 s. Because this stage was the middle period of the second motion cycle, the roll angle was close to the maximum value and the robot experienced greater deceleration (i.e., the deceleration process after reaching pre-set speed). If the load is too large (more than 20 kg), the target torque will increase and the selected drive module thus cannot provide enough torque to track the planned trajectory; however, the robot will gradually tend to the balance state at a constant walk speed. Furthermore, a fluctuation signature after reaching a pre-set speed can be avoided by planning a deceleration trajectory.

From the above analysis, it can be concluded that the crawling pattern generator with trunk swaying and FFC proposed in this paper shows an obvious improvement in stability during crawling motion at different speeds, and the experiments performed with different loads show that the planar quadruped robot with TP legs can crawl stably under a load of at least 20 kg.

### 4.2. Experiment and Discussion

**Figure 14:**
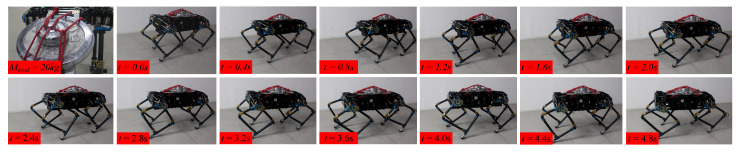
Snapshots of crawling motion with a 20 kg load during the third complete cycle (0.68 km/h).

To provide further verification of the feasibility of the crawling pattern generator proposed in this paper, we performed verification experiments on a planar quadruped robot platform that was developed independently. Based on the simulation results, crawling motion at 0.68 km/h with a 20 kg load was carried out using the physical robot. Figure 14 shows the photographs of the crawling process; the quadruped robot was able to move steadily straight forward in accordance with the planned trajectory.

**Figure 15:**
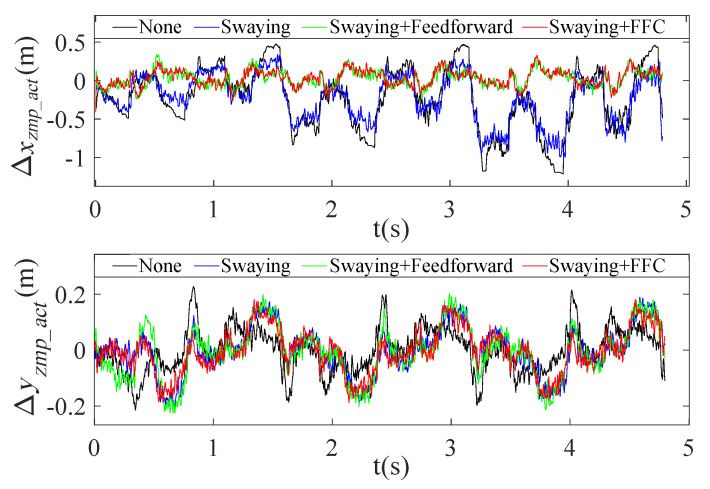
Actual ZMP tracking errors in crawling motion at the speed of 0.68 km/h with a 20 kg load.

The actual ZMP tracking errors in the crawling process are presented in Figure 15, which shows that both $\Delta x_{zmp\_act}$ and $\Delta y_{zmp\_act}$ showed larger fluctuations (−1.2 m to 0.5 m and −0.22 m to 0.22 m, respectively) with the original planning trajectory and swaying only; however, they could be maintained between −0.2 m and 0.3 m ($\Delta x_{zmp\_act}$) and −0.16 m and 0.16 m ($\Delta y_{zmp\_act}$) during the actual crawling process when trunk swaying and FFC were added, as shown in Figure 15.

According to the analysis of the crawling experiments, our robot was able to achieve stable crawling with a load of more than 20 kg, and the proposed crawling pattern generator reduced the ZMP tracking errors effectively. The load capacity can be symbolized by the payload–weight ratio (i.e., the ratio of the maximal payload mass and the robot mass) [19], and the comparison data of the robots’ payload capacity in Table 2 were obtained based on the above definition. To evaluate the payload capacity of our planar quadruped robot, a payload capacity comparison of the existing motor-driven robots is presented in Table 2 [13], where, *M* is the robot’s mass and Mot is the payload–weight ratio.

When compared with the Minitaur, our robot is larger in size and has a higher load ratio (66%). Although other robots with lateral DOFs have larger self-weight ratios, the experimental results showed that our robot’s load ratio was more than twice that of the others, except for the SpotMini. In fact, the load ratio calculated in this paper was based on the driver module configuration selected. However, the load capacity can be further improved by optimizing the relationship between the mass of the driver module and its driving ability.

## 5. Conclusions

This paper proposed a structural design method and a novel crawling pattern generator for planar quadruped robots for high-payload locomotion. Based on the definiton of the leg types of the robot, the evaluation functions used to measure the legs’ load capacity were established, and the TP leg structure, which had the best load capacity, was selected through the quantitative analysis. A highly integrated design of the driver module was proposed, and its payload capacity and impact resistance were improved greatly. The mass ratio of the legs to the whole robot was 0.18, which made the motion model more accurate. Then, to improve the stability of crawling motion with a load, we proposed a crawling pattern generator that mainly included the realization of the lateral movement of the CoM based on trunk swaying and a crawling stability optimization approach that combined feedforward and feedback compensation control (FFC). Based on the results of the simulations and experiments performed at different speeds and loading conditions, we concluded that the robot can crawl stably when the load is greater than 66% of its own weight, which verifies the feasibility and the superiority of the high-payload quadruped robot. In future work, we will study high-payload locomotion control at higher speeds on uneven ground.

## Figures and Tables

**Table 1:** Parameters for simulation and experiment.

Symbol	Items	Values
DoF	Degrees of freedom	4 × 2
$M_{s}$	Total mass	30 kg
$M_{sl}$	Single leg mass	1.4 kg
*H*	Constant CoM height	0.55 m
$L_{body}$	Length of robot	0.8 m
$W_{body}$	Width of robot	0.45 m
$L_{s}$	Stride length	0.3 m
*h*	Step height	0.1 m
*T*	Walk cycle period	1.6 s
Step	Control period	0.002 s
$t_{s}$	Quadruped support phase time	*T*/10
$k_{1}$	State feedback gain	10.1586
$k_{2}$	State feedback gain	−51.9226
$k_{3}$	State feedback gain	−32.9633

**Table 2:** Payload capacity comparison of representative motor-driven robots.

Robot	Ours	Minitaur	SpotMini	MIT Cheetah	StarlETH	Anymal C
*M*(kg)	30	5	25	33	23	50
Mot(%)	66	40	56	24	16	20

## References

[1] Eckert P., Schmerbauch A.E.M., Horvat T., Söhnel K., Fischer M.S., Witte H., Ijspeert A.J. (2018). Towards rich motion skills with the lightweight quadruped robot serval—A design, control and experimental study. Adaptive Behavior. Int. Conf. Simul. Adapt. Behav..

[2] Eckert P., Ijspeert A.J. (2019). Benchmarking agility for multilegged terrestrial robots. IEEE Trans. Robot..

[3] Zhong B., Zhang S., Xu M., Zhou Y., Fang T., Li W. (2018). On a cpg-based hexapod robot: Amphihex-ii with variable stiffness legs. IEEE/ASME Trans. Mechatron..

[4] Raibert M.H., Tello E.R. (2007). Legged robots that balance. IEEE Expert..

[5] Neunert M., Stauble M., Giftthaler M., Bellicoso C.D., Carius J., Gehring C., Hutter M., Buchli J. (2018). Whole-body nonlinear model predictive control through contacts for quadrupeds. IEEE Robot. Autom. Lett..

[6] Gao J., Li M., Li Y., Wang B. (2017). Singularity analysis and dimensional optimization on a novel serial-parallel leg mechanism. Procedia Eng..

[7] Buchli J., Ijspeert A.J. (2008). Self-organized adaptive legged locomotion in a compliant quadruped robot. Auto. Robot..

[8] Raibert M., Blankespoor K., Nelson G., Playter R. (2008). BigDog, the Rough-Terrain Quadruped Robot. World Congress. Proc. World Congr. Int. Fed. Autom. Control..

[9] Semini C., Barasuol V., Goldsmith J., Frigerio M., Focchi M., Gao Y. (2016). Design of the hydraulically-actuated, torque-controlled quadruped robot HyQ2Max. IEEE/ASME Trans. Mechatron..

[10] Yang K., Rong X., Zhou L., Li Y. (2019). Modeling and analysis on energy consumption of hydraulic quadruped robot for optimal trot motion control. Appl. Sci..

[11] Hutter M., Gehring C., Jud D., Lauber A., Bellicoso C.D., Tsounis V., Hwangbo J., Bodie K., Fankhauser P., Bloesch M. ANYmal—A highly mobile and dynamic quadrupedal robot. Proceedings of the 2016 IEEE/RSJ International Conference on Intelligent Robots and Systems (IROS).

[12] Bledt G., Powell M.J., Katz B., Carlo J.D., Wensing P.M., Kim S. MIT Cheetah 3: Design and Control of a Robust, Dynamic Quadruped Robot. Proceedings of the 2018 IEEE/RSJ International Conference on Intelligent Robots and Systems (IROS).

[13] Kenneally G., De A., Koditschek D.E. (2016). Design principles for a family of direct-drive legged robots. IEEE Robot. Autom. Lett..

[14] Kau N., Schultz A., Ferrante N., Slade P. Stanford doggo: An open-source, quasi-direct-drive quadruped. Proceedings of the 2019 International Conference on Robotics and Automation (ICRA).

[15] Arm P., Zenkl R., Sun B., Dietsche A., Hinder J. SpaceBok: A Dynamic Legged Robot for Space Exploration. Proceedings of the 2019 International Conference on Robotics and Automation (ICRA).

[16] Gagliardini L., Tian X., Gao F., Qi C., Chevallereau C., Zhao X. Modelling and Trajectory Planning for a Four Legged Walking Robot with High Payload. Proceedings of the International Conference on Social Robotics.

[17] Chen X., Gao F., Qi C., Zhao X. Spring parameters design to increase the loading capability of a hydraulic quadruped robot. Proceedings of the International Conference on Advanced Mechatronic Systems.

[18] Xin G., Zhong G., Deng H. Dynamic analysis of a hexapod robot with parallel leg mechanisms for high payloads. Proceedings of the Asian Control Conference.

[19] Huang Z., Xu W., Wang Z., Mu Z. The design, control and experiment of a high payload-weight hexapod robot. Proceedings of the IEEE International Conference on Robotics and Biomimetics. (ROBIO).

[20] Mcghee R.B., Frank A.A. (1968). On the stability properties of quadruped creeping gaits. Math. Biosci..

[21] Vukobratovic M., Borovac B. (2004). Zero-moment point–thirty five years of its life. Int. J. Humanoid Robot..

[22] Kajita S., Kanehiro F., Kaneko K., Fujiwara K., Hirukawa H. Biped walking pattern generation by using preview control of zero-moment point. Proceedings of the 2003 IEEE International Conference on Robotics and Automation (Cat. No.03CH37422).

[23] Yu Z., Zhou Q., Chen X., Li Q., Meng L., Zhang W., Huang Q. (2018). Disturbance rejection for biped walking using zero-moment point variation based on body acceleration. IEEE Trans. Ind. Electron..

[24] Yuan S., Zhou Y., Luo C. Crawling Gait Planning Based on Foot Trajectory Optimization for Quadruped Robot. Proceedings of the 2019 IEEE International Conference on Mechatronics and Automation (ICMA).

[25] Chen K., Ha S., Yamane K. Learning Hardware Dynamics Model from Experiments for Locomotion Optimization. Proceedings of the 2018 IEEE/RSJ International Conference on Intelligent Robots and Systems (IROS).

[26] Xu J., Lang L., An H., Ma H., Zhu K. Compliance control based for a quadruped robot walking over rough terrain. Proceedings of the 2015 Chinese Automation Congress (CAC).

[27] Murray R.N., Li Z., Sastry S. (1994). A Mathematical Introduction to Robotics Manipulation.

[28] He Z., Meng F., Fan X., Kang R., Huang Q. Development of A Parallel-elastic Robot Leg for Loaded Jumping. Proceedings of the International Conference on Advanced Robotics and Mechatronics, (ICARM).

[29] Kagami S., Nishiwaki K., Sugihara T., Inoue H. Design and implementation of software research platform for humanoid robotics: H6. Proceedings of the 2001 ICRA. IEEE International Conference on Robotics and Automation (Cat. No.01CH37164).

